# Radical prostatectomy outcomes in renal transplant recipients: a retrospective case series of Thai patients

**DOI:** 10.1186/s12894-021-00862-z

**Published:** 2021-07-06

**Authors:** Kun Sirisopana, Pocharapong Jenjitranant, Premsant Sangkum, Kittinut Kijvikai, Suthep Pacharatakul, Charoen Leenanupunth, Wachira Kochakarn, Wisoot Kongchareonsombat

**Affiliations:** 1 Division of Urology, Department of Surgery, Faculty of Medicine, Ramathibodi Hospital, Mahidol University, 270 Thanon Rama VI, Thung Phaya Thai, Ratchathewi, Bangkok, 10400 Thailand; 2 Division of Urology, Department of Surgery, Police Hospital, Bangkok, Thailand

**Keywords:** Prostate cancer, Radical prostatectomy, Laparoscopy, Robot-assisted laparoscopic surgery, Renal transplant recipients

## Abstract

**Background:**

The incidence of prostate cancer in renal transplant recipients (RTR) is similar to the general population. Radical prostatectomy (RP) is the standard of care in the management of clinically localized cancer, but is considered complicated due to the presence of adhesions, and the location of transplanted ureter/kidney. To date, a few case series or studies on RP in RTR have been published, especially in Asian patients. This study aimed to evaluate the efficacy and safety and report the experience with RP on RTR.

**Methods:**

We retrospectively reviewed data of 1270 patients who underwent RP from January 2008 to March 2020, of which 5 patients were RTR. All available baseline characteristics, perioperative and postoperative data (operative time, estimated blood loss (EBL), complications, length of hospital stay, complication), pathological stage, Gleason score, surgical margin status, and pre/postoperative creatinine were reviewed.

**Results:**

Of the 5 RTR who underwent RPs (1 open radical prostatectomy (ORP), 1 laparoscopic radical prostatectomy (LRP), 2 robotic-assisted laparoscopic radical prostatectomies (RALRP), and 1 Retzius-sparing RALRP (RS-RALRP)) prostatectomy, the mean age (± SD) was 70 (± 5.62) years. In LRP and RALRP cases, the standard ports were moved slightly medially to prevent graft injury. The mean operative time ranged from 190 to 365 min. The longest operative time and highest EBL (630 ml) was the ORP case due to severe adhesion in Retzius space. For LRP and RALRP cases, the operative times seemed comparable and had EBL of ≤ 300 ml. All RPs were successful without any major intra-operative complication. There was no significant change in graft function. The restorations of urinary continence were within 1 month in RS-RALRP, approximately 6 months in RALRP, and about 1 year in ORP and LRP. Three patients with positive surgical margins had prostate-specific antigen (PSA) persistence at the first follow-up and 1 had later PSA recurrence. Two patients with negative margins were free from biochemical recurrence at 47 and 3 months after their RP.

**Conclusions:**

Our series suggested that all RP techniques are safe and feasible mode of treatment for localized prostate cancer in RTR.

## Background

Along with the improvement in renal transplantation techniques and post-transplantation care, the recipients live longer. Most renal transplant recipients (RTR) are aged older than 45 years [1], so prostate cancer screening and treatment are still important in these patients.

Prostate cancer is the fifth most common cancer in Thai men [2], and the number of cases continues to increase despite active screening. Unfortunately, the incidence of prostate cancer in RTR is similar to the general population [3]. Radical prostatectomy (RP) is the standard of care in the management of clinically localized cancer. Radical prostatectomy in RTR is considered complicated due to the presence of adhesions or the location of transplanted ureter/kidney. Radiotherapy, active surveillance, or watchful waiting are alternative options. However, radiotherapy is less recommended due to post-radiation complications. To date, there have been a few case series or studies on radical prostatectomy in renal transplant recipients (RTR) published [4–9], especially in Asian patients. This case series aimed to demonstrate our experience with RP for localized prostate cancer in RTR and evaluated the surgical and oncological outcomes.

## Methods

### Study Design and population

We retrospectively reviewed the data of 1270 patients who underwent RP from January 2008 to March 2020 at Ramathibodi Hospital, Bangkok, Thailand. There were 5 patients (0.39%) who underwent renal transplantations before RP. The RP approaches in those RTR patients were open radical prostatectomy (ORP) in 1 case, laparoscopic radical prostatectomy (LRP) in 1 case, Retzius-sparing robotic-assisted laparoscopic radical prostatectomy (RS-RALRP) in 1 case and robotic-assisted laparoscopic radical prostatectomy (RALRP) in 2 cases. The Committee for Research of the Faculty of Medicine, Ramathibodi Hospital, Mahidol University approved the study prior to commencing the study data review and collection (Approval certificate ID MURA2020/298). Individual informed consent was exempted by the committee due to the type of the research. The study adhered to STROBE guidelines. The principles of the Helsinki Declaration were followed during the study data collection, and the confidentiality of the patients’ data was guaranteed.

### Surgical techniques

**Fig. 1:**
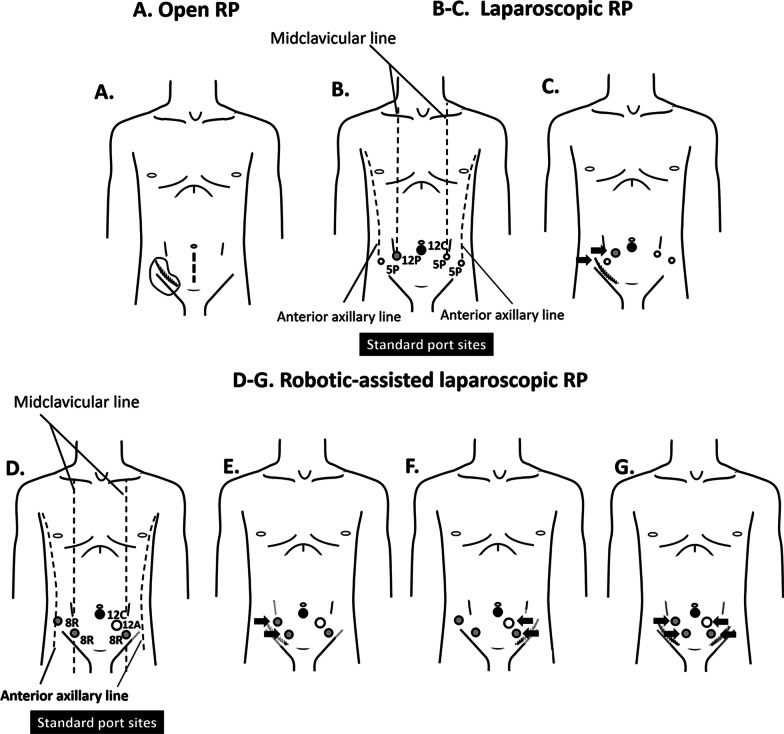
Skin incision and port placement for the radical prostatectomy (RP). **A** Skin incision (dash line) of open RP in right-sided renal transplantation; **B** Laparoscopic port placement in general population; **C** Laparoscopic port placement in right-sided renal transplantation; **D** Robotic port placement in general population; **E–G** Robotic port placement in right-sided (**E**), left- sided (**F**), and both sides renal transplantation (**G**). Symbols: 12C,12 mm camera port; 12P, 12 mm laparoscopic port; 5P, 5 mm laparoscopic port; 8R, 8 mm robotic port, 12A, 12 mm assistant port, black arrow; direction of port modification/adjustment

We performed ORP in a retropubic fashion through a low midline incision (Fig. 1A) according to our regular ORP approach [10]. Briefly, the space of Retzius was developed using blunt and sharp dissection along the outside of the left umbilical ligament. At this step, special care was taken to avoid injury to the transplanted ureter and dissected only along the left umbilical ligament. Endopelvic fascia on both sides was bluntly opened, the puboprostatic ligament was dissected. The dorsal venous complex was sutured and ligated with Vicryl No.1. The bladder neck was incised with monopolar cautery, followed by pulling and traction of foley’s catheter, dissection of seminal vesicle and vas deferens, and opening Denonvilliers' fascia. At the moment, the posterior surface of the prostate was freed. The lateral prostatic pedicles were dissected with monopolar cautery and ligated with non-nerve sparing, followed by incision of the urethra using Metzenbaum scissors. We performed vesicourethral anastomosis with interrupted sutures, using Vicryl No. 3/0, 6 sutures. Before the last suture, a new 20 Fr Foley’s catheter insertion was performed through the urethra into bladder. The surgical areas were examined to ensure there is no active bleeding, followed by Silastic drain placement in the cul-de-sac.

The LRP was performed in an extraperitoneal fashion using 5 trocars with some modification from the standard port positions [10] to facilitate graft injury prevention (Fig. 1B, C). Firstly, we incised sub-umbilical and created extraperitoneal space with kidney shape balloon with a dissection balloon (PDB, Covidien, United States). Extra-pneumoperitoneum was developed by CO_2_ insufflation to create an abdominal pressure of 15 mmHg, followed by the modified port placement as Fig. 1C, and trocar insertion under direct visualization. The patients were placed in thirty-degree standard Trendelenburg position with cushioning for the dependent zone. The Retzius space was carefully developed to avoid injury to the transplanted ureter. Endopelvic fascia on both sides was opened, followed by puboprostatic ligament dissection and bladder neck incision with monopolar cautery. We controlled the dorsal venous complex with Vicryl No.1 CT-1 needle. After that, the Foley’s catheter was pulled to traction, followed by dissection of seminal vesicle and vas deferens and opening Denonvilliers' fascia to free the posterior surface of the prostate, respectively. We controlled the lateral prostatic by Hem-o-lock clip and dissected it with a vessel sealing device (LigaSure Impact Curved, Large jaw, Medtronic, United Kingdom) with non-nerve sparing, followed by urethra incision with cold scissors. We performed vesicourethral anastomosis with continuous watertight sutures with Vicryl 3/0. To ensure adequate insertion of 20 Fr Foley’s catheter into the urinary bladder, we inserted the catheter before passing the anterior stitch. After completing the anastomosis, a closed suction drain was placed in the cul-de-sac. The specimen was retrieved through a sub-umbilical incision using a laparoscopic bag.

The RALRPs were performed using the Da Vinci Si Surgical System by transperitoneal technique with 5 trocars. In general, the procedures of RALRP in RTR were not different from RALRP in general patients but the trocars were placed slightly medially (Fig. 1E, F, G) from the standard port sites [10] (Fig. 1D) to deliver sufficient access without graft injury. Veress needle is punctured at the sub-umbilical area to establish pneumoperitoneum. of 15 mmHg. A 12 mm sub-umbilical trocar was inserted into abdominal space to be used as a camera port to insert the resting trocar under direct visualization. On the right side, two robotic trocars were placed with at least 8 cm apart from each other (Arm 1, Arm 3). For the left side, a robotic trocar was placed (Arm 2), and the assistance 12 mm trocar was placed between the robotic port and the camera port. Robotic Arm 1, 2, and 3 were equipped with Monopolar scissors, Bipolar Maryland, and ProGrasp forceps, respectively. The patients were placed in thirty-degree standard Trendelenburg position with cushioning for dependent zone. A Retzius space was developed carefully to avoid injury to the transplanted ureter. Endopelvic fascia on each side was opened, followed by the puboprostatic ligament dissection. We controlled the dorsal venous complex using barbed suture No.1 (V-Loc PBT wound closure device, Medtronic, United Kingdom). The bladder neck incision was performed with monopolar and bipolar cautery then Foley’s catheter was gently pulled to traction, followed by dissection of seminal vesicle and vas deferens, and opening Denonvilliers' fascia to free the posterior surface of prostate. The lateral prostatic pedicles were controlled with Hem-o-lock clip and dissected with monopolar and bipolar cautery. One case was performed with inter-fascial nerve-sparing and another case was non-nerve sparing. The urethra was then incised by cold scissors. A vesicourethral anastomosis with continuous watertight sutures, using barbed suture 3/0 (V-Loc PBT wound closure device, Medtronic, United Kingdom) was performed. The procedures for Foley’s catheter insertion and specimen retrieval were the same as LRP.

The RS-RALRP was performed using the Da Vinci Si Surgical System with 5 trocars. A paraumbilical incision was made. The first 12-mm camera trocar was placed with the use of an open Hasson technique. CO_2_ was insufflated to obtain pneumoperitoneum up to the level of 15 mmHg, and 2 other robotic trocars were placed in the left and right iliac fossae under direct visualization. The trocar in the right iliac fossa was inserted cautiously to avoid injury to graft. A 12-mm assistant port was placed in the left iliac fossa. The patient was placed in thirty-degree standard Trendelenburg position with cushioning for dependent zone. At the anterior surface of the Douglas space, parietal peritoneum was incised. Seminal vesicles and vas deferens were dissected. Denonvilliers' fascia was opened by the posterolateral surface of the prostate in an antegrade direction, reaching the prostatic apex. The bladder neck was incised. The anterior surface of the prostate was bluntly dissected from the dorsal venous complex without incision, followed by the incision of urethra with cold scissors. The vesicourethral anastomosis was performed with continuous watertight sutures, using barbed suture 3/0 (V-Loc PBT wound closure device, Medtronic, United Kingdom). Before passing the posterior stitch, the 20 Fr Foley’s catheter was passed into the bladder and the anastomosis was completed. A closed suction drain was placed in the prevesical space. The specimen was retrieved with the use of laparoscopic bag through a paraumbilical incision. The peritoneum at the Douglas space was closed.

All RPs were done by 2 experienced senior instructor surgeons without pelvic lymphadenectomy. The surgeons and patients worked and made decisions together to select the procedure techniques.

### Baseline characteristics and preoperative parameters

The patients’ age, body weight (kgs), height (cms), body mass index (BMI), Gleason score (GS) of the biopsy specimen, initial prostate-specific antigen (PSA) level, clinical T stage (TNM classification), prostate cancer risk group (according to the National Comprehensive Cancer Network [NCCN] Clinical Practice Guidelines in Oncology: Prostate Cancer Version 2, 2020) [11], graft localization, type of donor and time from transplantation to RP were collected from the patients’ medical records, including histo-pathological reports, and imaging study reports.

### Surgical outcomes

Surgical outcomes included type of procedure, undergoing nerve-sparing, operative time, estimated blood loss (EBL), perioperative complications, including transfusion rate, adjacent organ injury of the bladder, rectum, ureter, bowel, or blood vessel, length of hospital stay (days, which were determined by subtracting the date of admission from the date of discharge) and post-operative complication (Clavien-Dindo classification).

### Oncological outcomes

The prostate weight (g), GS of the specimen, pathological T stage, and the margin status were evaluated by an experienced uropathologist in accordance with the NCCN guidelines. A positive surgical margin (PSM) was simply defined as cancer cells extending to the inked surface of the specimen [12]. The follow-up period and post-operative course were collected from medical records.

### Statistical analysis

Sample size calculation was not applied. All RTR patients, who underwent RP, were included in the study. Descriptive statistics, i.e. frequency with proportion or percentage, mean with SD, and range, were used to present the study data. There were no comparisons between RP approaches due to small sample size. Statistical analysis was performed using StataSE, version 20 (IBM, New York, NY, USA).

## Results

### Baseline characteristics

Five patients were included in this study. The baseline characteristics and preoperative parameters are demonstrated in Table 1. The mean patients’ age at the time of RP (± SD) was 70 (± 5.62) years (ranged from 64–79 years). Risk group stratification in these patients was low (1/5), favorable intermediate (1/5), unfavorable intermediate (1/5), high risk (1/5) and very high risk (1/5). The kidney allografts were implanted in the right iliac fossa in the first 3 cases (Case 1, 2, and 3), in the left iliac fossa in the 4th case (Case 4), and the last case (Case 5) had renal grafts on both sides of iliac fossa. The first 2 cases and Case 4 received kidney from deceased donors, Case 3 from living related donor and Case 5 from deceased donor for the left side and living related donors for the right side. There was no adjustment in the immunosuppressive regimen during the surgery, but a watchful follow-up by nephrologist was performed for the purpose of early detection of complication. Table 1 presents the patients’ baseline characteristics.

**Table 1:** Baseline characteristics

	Case 1	Case 2	Case 3	Case 4	Case 5
Age (years)	67	64	74	66	79
BMI (kg/m^2^)	26.99	22.86	29.92	23.88	28.21
Biopsy Gleason score	4 + 5	3 + 3	3 + 4	4 + 4	3 + 3
Preoperative PSA (ng/ml)	25.66	10.84	11.53	130	9.63
Clinical T stage	T3b	T1c	T1c	T3a	T1c
NCCN Prostate cancer risk group	Very high	Favorable intermediate	Unfavorable intermediate	High	Low
Graft localization	Right	Right	Right	Left	Both sides
Donor	DDKT	DDKT	LRKT	DDKT	DDKT and LRKT
Time from transplantation to RP (years)	13	9	21	8	13

BMI, body mass index; DDKT, deceased-donor kidney transplantation; LRKT, living-related kidney transplantation; NCCN, National Comprehensive Cancer Network; PSA, prostate-specific antigen; RP, radical prostatectomy

### Surgical outcomes

The RP approaches were performed with non-nerve sparing, except for Case 5 that performed RP with unilateral nerve-sparing. The surgical data and outcomes were demonstrated in Table 2. The mean operative time (± SD) was 237 (± 64.47) minutes (ranged from 190 to 365 min). The longest operative time was Case 1 who underwent ORP (365 min) with the EBL of 630 mls due to severe adhesion in Retzius space. For LRP (210 min) and RALRP (210, 190, and 210 min), the operative times seemed comparable.

**Table 2:** Intraoperative, post-operative and oncological outcomes

	Case 1	Case 2	Case 3	Case 4	Case 5
Operative date	23-Nov-05	11-June-10	08-Apr-15	06-Dec-18	05-Mar-20
Type of RP	ORP	LRP	RS-RALRP	RALRP	RALRP
Nerve sparing	No	No	No	No	Unilateral
Operative time (min)	365	210	210	190	210
EBL (mLs)	630	300	250	150	100
Prostate specimen weight (g)	34.8	40	24	23.5	30
Perioperative complication	None	None	None	None	None
Complication by Clavien-Dindo classification	2	2	1	1	1
Complication	Blood transfusion and postoperative fever	Blood transfusion and postoperative fever			
LOS (days)	13	6	8	5	7
Pathological Gleason score	4 + 5	3 + 3	4 + 3	4 + 5	3 + 4
Pathological T stage	T3b	T2a	T2c	T3b	T2a
Marginal status	Positive	Negative	Positive	Positive	Negative
Follow-up period (months)	129	47	63	31	6
*Creatinine (mg/dl)*					
Preoperative	1.3	1	1.41	0.66	1.11
1 day postoperative	1.2	0.8	1.38	0.57	0.87
3 days postoperative	1.12	Not measured	0.88	0.58	0.79
7 days postoperative	1.25	Discharged	1.06	Discharged	0.78
The last available Cr	1.6 Year 2016	1.28 Year 2014	1.74 Year 2020	0.65 Year 2020	1.05 May-20
Survival/oncological status	PSA persistence, on ADT until death in 2016 due to progressive allograft dysfunction with uremia	Recurrence free until death in 2014 due to sigmoid colon carcinoma with gut obstruction and septic shock	PSA recurrence at 13 months. Then on ADT, PSA < 0.003 at the follow up on 9 June 2020	PSA persistence, on ADT. PSA of 0.85 at the follow-up on 25 May 2020	Recurrence free at the follow-up on May 2020
Return of urinary continence after surgery	1 year	1 year	1 month	6 months	6 months

ADT, androgen deprivation therapy; Cr, creatinine; EBL, estimated blood loss; LRP, laparoscopic radical prostatectomy; LOS, length of hospital stay; ORP, open radical prostatectomy; PSA, prostate-specific antigen; RALRP, robotic-assisted laparoscopic radical prostatectomy; RP, radical prostatectomy; RS-RALRP, Retzius-sparing robotic-assisted laparoscopic radical prostatectomy

There was no perioperative complication in all 5 patients. The postoperative course of the patients was uneventful, excepted for Case1 and Case 2 that required blood transfusion, due to anemia and postoperative fever in post-operative day 1 which subsided in the next day.

In Case 1 and 2, the urethral catheter was removed on post-operative day 13 and 14, respectively after cystogram while the others were removed on post-operative day 7. The differences in urethral catheter retention duration depended on the surgeon's confidence, but was not from complication. In the first and second case of RP in RTR patients (Case 1 and Case 2), the surgeon decided to retain the catheter for approximately two weeks post-operation to ensure the anastomosis healing from the open and laparoscopic complexity. In the latter cases (Cases 3, 4, and 5), the surgeon decided to retain the catheter for 1 week after RP because they were confident that one week was sufficient to secure the anastomosis after RP using the Da Vinci Si Surgical System. The restorations of urinary continence after surgery were within 1 month in RS-RALRP (Case 3), approximately 6 months in RALRP (Case 4 and 5), and about 1 year in ORP (Case 1) and LRP (Case 2).

Wound related complication was not encountered in all patients. Means length of hospital stay (± SD) was 7.8 (± 2.79) days (ranged from 5 to 13 days). The longest hospital stay (13 days) was Case 1 who was the first case in our experience.

### Oncological outcomes

The oncological outcomes were also demonstrated in Table 2. Pathological analysis revealed GS 3 + 3 (1/5), 3 + 4 (1/5), 4 + 3 (1/5) and 4 + 5 (2/5). The pathological tumor stage (pT) was T2a (2/5), T2c (1/5) and T3b (2/5). Three patients had positive surgical margin, consisting of Case 1 who underwent ORP, had pathological GS of 4 + 5 and pT3b, Case 3 who underwent RS-RALRP, had the GS of 4 + 3 and pT2c, and Case 4 who underwent RALRP, had the GS of 4 + 5 and pT3b.

The follow-up period of the 5 patients ranged from 6 to 129 months. Case 1 (GS 4 + 5, pT3b and margin positive disease) had PSA persistence without distant metastasis and was treated with androgen deprivation therapy (ADT) until the patient died in 2016 from progressive allograft dysfunction with uremia (total follow-up period 129 months). Case 2 (GS 3 + 3, pT2a and negative margin) had remained recurrence free with total follow-up period of 47 months, until 2014, when the patient presented at hospital with the sigmoid colon cancer with gut obstruction and died from septic shock. Case 3 (GS 4 + 3, pT2c and margin positive disease) experienced a biochemical recurrence at 13 months post-operation. The patient was treated with ADT without adjuvant radiation therapy due to worrisome complication of radiation and remained on ADT, with undetectable PSA (follow-up period 63 months). Case 4 (GS 4 + 5, pT3b and margin positive disease) encountered PSA persistence without distant metastasis and was treated with ADT alone and remain on ADT, with PSA level of 0.85 (follow-up period 31 months). Case 5 (GS 3 + 4, pT2a and negative margin) had remained recurrence free with the total follow-up of 6 months. Additionally, graft function as represented by the serum creatinine level was stable before, during and after surgery in all patients as shown in Table 2.

## Discussion

Although improving in transplantation techniques and post-transplantation care, cancer remains a major adverse feature. The prevalence of prostate cancer in RTR is similar to the general population [3]. However, some studies showed the prevalence of genitourinary cancer is the second most common cancer in RTR [13], especially prostate cancer as the fifth most common cancer [14] with the prevalence range from 0.72 to 3.1% [7, 9]. In Thailand, the incidence of prostate cancer has been increasing and impactful as the fifth most common cancer in Thai men [2].

The prognosis and natural history of prostate cancer in RTR are not associated with worse outcomes than non-RTR. The standard treatments should be proposed to this population with satisfying surgical and oncological outcomes [15, 16]. There are several treatments for prostate cancer, including active surveillance, RP, radiotherapy, hormonal therapy, and watchful waiting. Each option is viable and should be a shared decision with patients.

In our experience, we do not believe that RP should be avoided in selected patients even with the history of abdominal surgery. Some previous literature revealed that prior abdominal surgery does not impact surgical outcomes and complications [17]. Even, there are potentially risks of injury due to smaller working space, adhesion, and transplanted renal and ureter, the surgery itself is possible. Moreover, a systematic review also showed that RP is the preferred treatment of localized prostate cancer [15].

Despite all these difficulties, multiple approaches of radical prostatectomy, including retropubic [18, 19], perineal [20], laparoscopic [21–23] and robotic-assisted [6, 24, 25] have been used in the treatment of prostate cancer in RTR, with comparable outcomes. Classically, radical retropubic prostatectomy was performed and obtained good results, although a study reported a graft injury event during retraction [26]. In our study, we performed retropubic ORP with partial bladder mobilization only along contralateral side of the umbilical ligament to avoid injury to the transplanted ureter. The perineal approach is also a viable procedure with some edge, including less manipulation of transplanted kidney and ureter [20]. However, we did not use this approach in our series. In Shah et al. [27] reported the first case of LRP in localized prostate cancer in RTR. In our experience, we used the extraperitoneal approach with medial port insertion from the standard port placement to avoid injury to transplanted graft and ureter.

Recently, the robotic-assisted procedure has become more routinely used. In Jhaveri et al. [28] presented the first RALRP in RTR with technical adjustment to mitigate graft injury. In our series, the surgical modification includes slight medial movement of trocars placement with an assistant port on contralateral side to renal graft, initiating the Retzius space dissection from the contralateral side with limited ipsilateral dissection, and constant awareness of vesicoureteral anastomosis to avoid injury to transplant graft. However, Wagener et al. [29] suggested that routine placement of trocar was also feasible. In Mistretta et al. [9] reported the use of RS-RALRP in RTR with modification of 12 mm assistant port to provide more medial and cranial respect to the standard set to avoid injury to graft. We performed RS-RALRP with medial placement of 5 trocars similar to RALRP and also preferred this approach more than others due to the anatomical safety. Additionally, if severe tension is encountered when performing vesicourethral anastomosis, we suggest using continuous watertight sutures by including some pelvic floor muscle into the stitch to prevent cutting through surrounding organ/tissues.

To cope with the difficulties for vesicourethral anastomosis, we performed the anastomosis by continuous watertight suturing technique with V-Loc*™* in RALRP and RS-RALRP. The anastomosis was quite simple in RALRP and RS-RALRP, because the Da Vinci Surgical System provided more visualization and accessibility to the suturing area to meticulously handle the anastomosis. In LRP, the same anastomosis technique as RALRP and RS-RALRP was performed, but it took a longer duration due to less clear visualization of the operative field and more difficulty to handle the suturing. In ORP, we used the parachute technique with Vicryl 3/0, 6 stitches for the anastomosis. The results from the present study showed that the patient who underwent RS-RALRP or RALRP was more likely to regain urinary continence earlier than those who underwent ORP or LRP.

In our series, all RPs in RTR were performed without lymphadenectomy to keep the procedure to be generally simple and less time consuming. In addition, the lymphadenectomy can cause collateral damage to the vascular and ureterovesical anastomosis of the transplanted kidney. The lymphadenectomy also does not provide better overall survival outcomes [30]. Although this procedure may be required in some cases, we suggest evaluating each patient individually based on the future need for a second transplant.

In terms of marginal status, there were 3 out of 5 patients having PSM. Two PSM patients (Case 1 and Case 4) were high and very high-risk group patients, while another patient (Case 3) was in the unfavorable intermediate-risk group. The patient Case 3 was our first RTR patient performing RS-RALRP and our early experience in RS-RALRP approach. The two negative surgical margin patients were favorable intermediate and low-risk group patients. The presence of PSM seemed to be likely related to the extent and severity of the disease.

Radiotherapy, including intensity-modulated radiation therapy or low dose rate prostate brachytherapy, is also feasible and acceptable minimally invasive treatment for selected RTR [31, 32]. However, some complications were encountered such as incontinence, urethral stricture, and obstruction of distal ureter, resulting in decreased graft function [33, 34]. There are several limitations of our study. We did not perform comparison among the RP approaches due to small number of patients. To our knowledge, our series demonstrated the first largest series in Thailand about RP in RTR that includes all different techniques. Multicenter studies were suggested in the future to confirm the safety of RP in RTR.

## Conclusions

The results from this series suggested that radical prostatectomy was a feasible and safe operation for the treatment of localized prostate cancer in renal transplant recipients. Moreover, minimally invasive techniques, especially robotic-assisted laparoscopic radical prostatectomy, provided advantage because they allowed greater visualization and handling of instruments in a restricted working space.

## Data Availability

All relevant (unidentifiable) data are within the manuscript. Additional patient data (unidentifiable) can be requested from the first or corresponding authors upon reasonable reason.

## Abbreviations

BMI: Body mass index
GS: Gleason score
LRP: Laparoscopic radical prostatectomy
NCCN: National Comprehensive Cancer Network
ORP: Open radical prostatectomy
PSA: Prostate-specific antigen
RALRP: Robotic-assisted laparoscopic radical prostatectomy
RP: Radical prostatectomy
RS-RALRP: Retzius-sparing robotic-assisted laparoscopic radical prostatectomy
RTR: Renal transplant recipients
